# Optimization of Spinal Reconstructions for Thoracolumbar Burst Fractures to Prevent Proximal Junctional Complications: A Finite Element Study

**DOI:** 10.3390/bioengineering9100491

**Published:** 2022-09-21

**Authors:** Chia-En Wong, Hsuan-Teh Hu, Yu-Heng Huang, Kuo-Yuan Huang

**Affiliations:** 1 Section of Neurosurgery, Department of Surgery, National Cheng Kung University Hospital, College of Medicine, National Cheng Kung University, Tainan 704, Taiwan; 2 Department of Civil Engineering, National Cheng Kung University, Tainan 701, Taiwan; 3 Department of Civil and Disaster Prevention Engineering, National United University, Miaoli 360, Taiwan; 4 Department of Orthopedics, National Cheng Kung University Hospital, College of Medicine, National Cheng Kung University, Tainan 704, Taiwan

**Keywords:** finite element, proximal junctional failure, spinal reconstruction, thoracolumbar

## Abstract

The management strategies of thoracolumbar (TL) burst fractures include posterior, anterior, and combined approaches. However, the rigid constructs pose a risk of proximal junctional failure. In this study, we aim to systemically evaluate the biomechanical performance of different TL reconstruction constructs using finite element analysis. Furthermore, we investigate the motion and the stress on the proximal junctional level adjacent to the constructs. We used a T10-L3 finite element model and simulated L1 burst fracture. Reconstruction with posterior instrumentation (PI) alone (U2L2 and U1L1+(intermediate screw) and three-column spinal reconstruction (TCSR) constructs (U1L1+PMMA and U1L1+Cage) were compared. Long-segment PI resulted in greater global motion reduction compared to constructs with short-segment PI. TCSR constructs provided better stabilization in L1 compared to PI alone. Decreased intradiscal and intravertebral pressure in the proximal level were observed in U1L1+IS, U1L1+PMMA, and U1L1+Cage compared to U2L2. The stress and strain energy of the pedicle screws decreased when anterior reconstruction was performed in addition to PI. We showed that TCSR with anterior reconstruction and SSPI provided sufficient immobilization while offering additional advantages in the preservation of physiological motion, the decreased burden on the proximal junctional level, and lower risk of implant failure.

## 1. Introduction

Burst fractures in the thoracolumbar (TL) spine are biomechanically characterized by the compression and failure of the anterior and middle spinal columns [1]. The management of TL burst fractures remains challenging, and different treatment strategies are available. These include posterior instrumentation (PI), anterior reconstruction, and three-column spinal reconstruction (TCSR) with combined PI and anterior reconstruction having been reported and deliberated on in the literature [2,3,4]. Among the different approaches, TCSR combined PI and anterior reconstruction with PMMA augmentation or titanium strut graft has been shown to provide immediate stabilization and restore spinal integrity in highly comminuted burst fractures [2]. Clinical studies have reported the advantages of TCSR over stand-alone PI or anterior-only surgery, including better neurological improvement, stability, restoration of sagittal balance, and less implant failure [2,3,4]. 

However, the rigid nature of the constructs increases the risk of adjacent segment complications. Adjacent compression fractures or adjacent disc degeneration at the proximal junctional level are devastating and result in proximal junctional failure (PJF) [5]. PJF can lead to spinal cord compression, spinal instability, and kyphotic deformity, which often require a second surgery. Reported risk factors for PJF include osteoporosis, older age, greater preoperative sagittal imbalance, and longer-segment fixation [6,7]. 

Compared to conventional long-segment PI (LSPI), which involves instrumentation at two levels above and below the index level, short-segment PI (SSPI) had less stiffness and less increase in stress on the adjacent levels but was associated with an increased risk of implant failure [8,9]. In contrast, other studies advocated that SSPI could provide sufficient stabilization [10]. Given the incongruent results, controversy remains in the choice of posterior fixation techniques [11,12]. Moreover, the complexity of TL reconstruction was increased by the different anterior vertebral column reconstruction materials, including Polymethyl methacrylate (PMMA) cement and titanium cages, which have both been widely used in vertebral body reconstruction [13]. Although previous studies have demonstrated similar clinical and radiographical outcomes between PMMA and titanium cages in TL reconstruction [14], their effects on the proximal junctional level and the biomechanics of PMMA and titanium cage-based reconstruction constructs have not been evaluated. Since PI resulted in the redistribution of the spinal loading between the anterior vertebral graft and the pedicle screw-rod construct [15], TCSR constructs involving combined anterior and posterior instrumentation should be evaluated as a whole. Given the biomechanical complexity of the TL region and the paucity of clinical and biomechanical evidence, the decision-making of selecting an optimal spinal reconstruction strategy remains controversial but appears to be important. 

To address the knowledge gap, we designed a finite element (FE) study to investigate the biomechanical performance of different TCSR constructs. Furthermore, we thought to find the optimal strategy to reduce the burden on the proximal junctional level. We established FE models of T10-L3 TL segments and the simulated failure of the L1 vertebral body to represent a burst fracture. Reconstructions with PI and TCSR constructs were simulated and compared. The range of motion (ROM) in flexion, extension, lateral bending, and axial rotation of the whole model and the reconstructed vertebra was analyzed. The mechanical burden of each construct on the reconstructed level, proximal junctional vertebra, disc, facets, and the construct itself was also compared. The objective of this study was aimed to compare and optimize the design of thoracolumbar reconstruction constructs by systematically investigating their biomechanical properties and how they affect the proximal junctional level. The knowledge gained from this study can provide help spine surgeons select an optimal TL reconstruction construct to minimize proximal junctional complications.

## 2. Materials and Methods

### 2.1. Generation of T10-L3 Finite Element Model 

**Figure 1:**
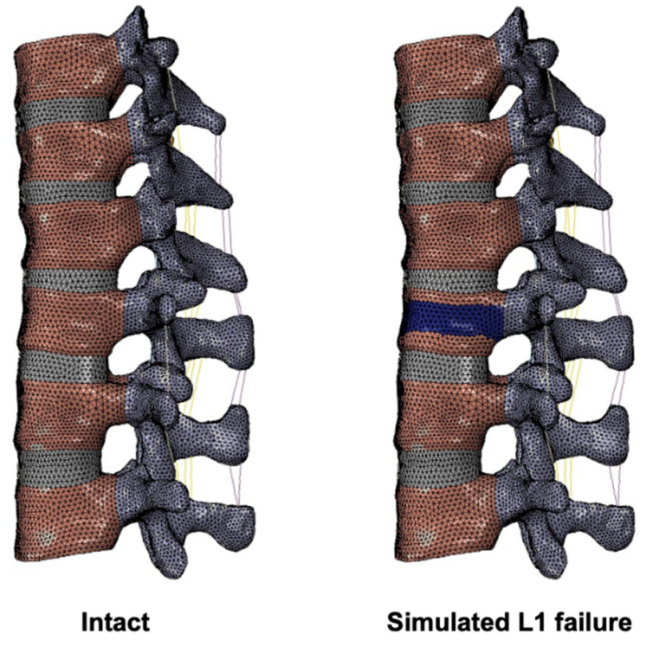
Finite element model of T10-L3 TL spine and simulation of L1 failure. The present finite element model of the intact spine (**left**) and simulated L1 failure (**right**). The weakened materials were indicated in blue.

A three-dimensional FE model of the T10-L3 thoracolumbar spine was created using 1 mm thin-cut axial computed tomography images obtained from a resin cast of an Asian male cadaver without spinal deformities or abnormalities (Figure 1). The images were imported into the software 3D-DOCTOR (Able Software Corp.) to reconstruct the geometric structure of the T10-L3 TL spine, and the corresponding mesh was prepared using the preprocessing software Patran (MSC Software). The mesh generation was performed with software Hypermesh (Altair Technologies Inc), and the FE models were imported into Abaqus 6.12 (Simulia Inc) to solve. In this study, we assumed linear and isotropic material properties for cancellous bone, cortical bone, posterior bony elements, endplate, and disc structures including annulus fiber layers, annulus ground substance, nucleus pulposus, and implant materials (Table 1). The material properties used in the present study were derived from the previous studies by Shin et al. and Wilcox et al. [16,17].

The model for a vertebra consisted of a vertebral body and a posterior element. For the vertebral body, a closed surface was first generated, consisting of cortical bones and endplates assigned to three-node shell elements (S3R). Considering the structures of the cortical bone and endplates of the vertebra, which cover the outer surface of the vertebral body and surround the cancellous bone, it is more reasonable to use shell elements than tetrahedral elements to represent the geometry of the cortex and endplates, and this modeling strategy was also reported in previous FE studies [18,19]. The thicknesses of the cortical bone and endplate were assigned as 0.35 mm and 0.5 mm, according to previous studies [20,21,22]

The interior of the cortical surface contained cancellous bone assigned to C3D4 continuum elements. The posterior element and the facet were modeled according to the original geometry using C3D4 tetrahedron elements as previously described [23,24]. A three-dimensional surface-to-surface contact with friction was assigned to simulate the facet contact behavior with a finite sliding interaction defined to allow random motions, including sliding, rotation, and separation. The friction characteristic was modeled with a classic isotropic Coulomb friction model with a friction coefficient of 0.1 [25].

The intervertebral discs (IVDs) were modeled with three different components: annulus fibers, annulus ground substance, and nucleus pulposus [25,26]. The IVDs were generated with the superior and inferior boundaries assigned to the endplates of the adjacent vertebra, and the outer boundaries of the IVDs were generated according to the scanned geometry. The annulus was constructed as a ring-shaped structure between the outer and inner annulus fibers. The annulus fibers were modeled with six layers of shell elements with a thickness of 1.5 mm. The annulus ground substance was defined between the two annulus fiber layers and was modeled by solid tetrahedral elements (C3D4). The nucleus pulposus was modeled by non-compressible solid tetrahedral linear elements (C3D4) inside the inner annulus fiber.

The ligamentous complex, including anterior longitudinal ligaments (ALL), posterior longitudinal ligaments (PLL), ligamentum flavum (LF), interspinous ligaments (ISL), and supraspinous ligaments (SSL), were modeled using hyperelastic, tension-only Truss elements (T3D2). The properties of the ligaments were adopted from Goel et al. [27]. The element types and number of elements used in the components of the spine are listed in Table 2.

### 2.2. Simulation of TCSR Models 

L1 burst fracture and vertebral body failure were simulated by weakening the material property of the middle 30% of the L1 vertebral body, according to the previously described method with some modifications (Figure 1) [28]. The Young’s modulus of the affected cortical and cancellous bone was decreased by 90% (Table 1). 

**Figure 2:**
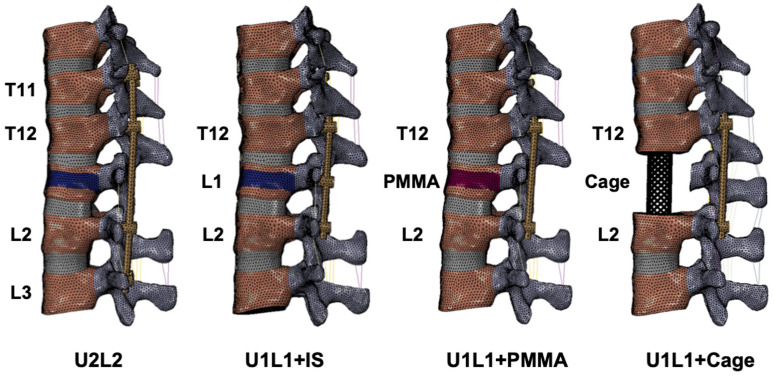
Different spinal reconstruction constructs. Four spinal reconstruction constructs simulated in this study including long-segment instrumentation (U2L2), short-segment instrumentation with intermediate screw (U1L1+IS), and TCSR constructs (U1L1+PMMA and U1L1+Cage). The weakened materials were indicated in blue, and PMMA was indicated in red.

For anterior reconstruction with PMMA, the surgery model was created by replacing the weakened elements in the L1 vertebral body with PMMA modeled by solid tetrahedral elements (C3D4) (Figure 2). For reconstruction with a titanium cage, an L1 corpectomy was simulated by removing the entire L1 vertebral body and the adjacent intervertebral discs at T12-L1 and L1-L2, and a titanium cage implant was simulated (Figure 2). The three-dimensional structures of the screws, rods, and titanium cage were created in the software Patran (MSC Software). The primary dimensions (diameter, length) of the pedicle screws for the thoracic and lumbar vertebrae were 5.5 mm × 45 mm and 6.5 mm × 45 mm, respectively. The diameter of the rods was 6 mm. The outer diameter, thickness, and height of the titanium cage were 14 mm, 2 mm, and 60 mm, respectively. The pedicle screws, rods, and cage were composed of titanium. The material properties of the implants were shown in Table 1. Mesh structures were prepared using the software Hypermesh 11.0 (Altair Technologies Inc., Fremont, CA, USA) and imported into Abaqus 6.12 (Simulia Inc., Johnston, RI, USA) to solve. 

Four surgery models were simulated in the present study, including (1) posterior fixation with LSPI alone (U2L2); posterior fixation with SSPI and intermediate pedicle screw at the L1 level (U1L1+IS); (3) TCSR with PMMA and SSPI (U1L1+PMMA); (4) TCSR with a titanium cage and SSPI (U1L1+Cage) (Figure 2).

### 2.3. Loading and Boundary Conditions 

The preload was set to 150 N and applied evenly using the follower load technique on the T10 superior endplate to simulate the weight of the upper body. For the simulation of the upper body weight, a preload ranging from 100–200 N was used in the literature, and a 150 N preload was chosen in the present study [29]. A 10 Nm moment was applied in the sagittal, coronal, and transverse plane to create motions in flexion–extension, lateral bending, and axial rotation, respectively. The boundary condition of the simulations was set with the nodes on the inferior endplate of L3 constrained in all directions. The interfaces between the bone, pedicle screws, PMMA, and titanium cage were assigned with tie constraints.

### 2.4. Convergence Test 

Convergence tests were performed on the intact model. First, the displacement of a reference point at the center of the T10 superior endplate was measured under a 150 N axial preload. Four different amounts of elements, 190,432, 208,776, 309,217, and 555,384, were compared for the displacements. By setting the displacement of the T10 superior endplate in a model consisting of 1,199,183 elements as the reference value, the errors of the simulations with the total number of elements reduced were all within 4.9 percent. Next, the maximum von Mises stress in posterior elements under a 150 N axial preload was compared. Compared to the reference model, the error of the four models with the total number of elements reduced were all within 7.9 percent. For material stress, it is generally expected that the error may be greater than displacement in FE models [30]. In the present model, we selected the model consisting of 309,217 elements for the intact model based on the small relative displacement error of 2.84% and a von Mises stress error of 4.18%, with the element size ranging from 1.0 to 2.0 mm. The convergence tests were performed on the implant models, including the pedicle screws and titanium cage. For the titanium cage, a final model consisting of 134,203 elements with mesh sizes ranging from 0.4 to 0.6 mm was selected. The error was 3.96% compared to the reference model with 220,141 elements. For the pedicle screw, a final model with 13,927 elements and element size ranging from 0.1 to 1.0 mm was selected. The error was 0.92% compared to the 23,550 element reference.

## 3. Results

### 3.1. Model Validation 

**Figure 3:**
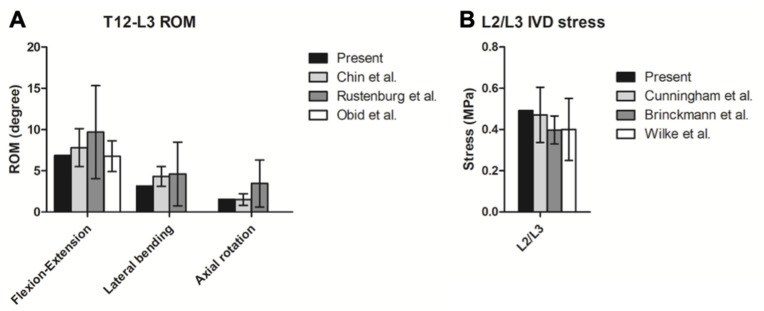
Validation of the present FE model. Comparisons between the (**A**) ROM and (**B**) IVD pressure of the present intact model with the literature [31,32,33,34,35,36] (presented in mean and standard deviation).

To validate the finite element model, the simulated ROM and IVD stress in the present intact T10-L3 model were compared with the literature. First, the ROM of the intact thoracolumbar model in flexion-extension, lateral bending, and axial rotation were compared with three in vitro experiments by Chin et al., Rustenburg et al., and Obid et al. [31,32,33]. The global ROM of the present intact model was as follows: flexion–extension, 6.86 degrees; lateral bending, 3.04 degrees; and axial rotation, 1.54 degrees. Compared with the literature, the results were all within one standard deviation (SD) (Figure 3A). Next, the intact model was also compared with the in vitro intradiscal pressure measurements at L2/L3 IVD conducted by Cunningham et al., Brinckmann et al., and Wilke et al. [34,35,36]. The maximal IVD stress of this model under sagittal flexion and extension was 0.49 Mpa, which was within one SD compared to the results of Cunningham et al. and Wilke et al. but slightly larger (1.24 SD) than the result reported by Brinckmann et al. (Figure 3B). An extended explanation of the differences is given in the Discussion section.

### 3.2. Global Range of Motion in the TL Spine 

**Figure 4:**
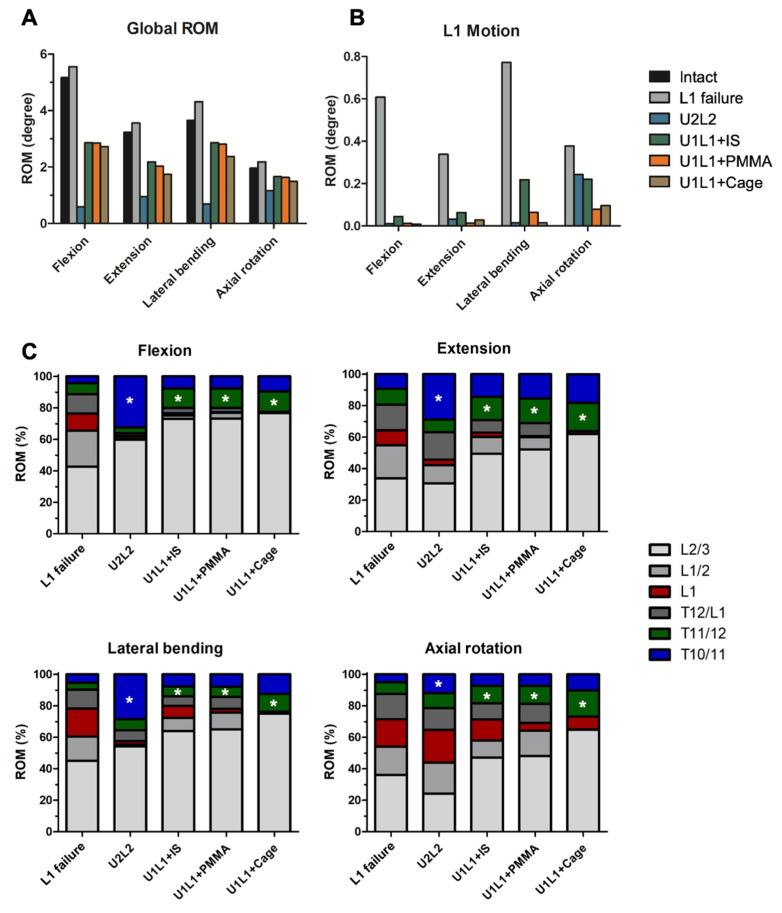
The global ROM, ROM in L1, and ROM distributions. The simulated global ROM (**A**) and the pathological ROM in the L1 vertebra (**B**). ROM distributions (**C**) among the T10–L3 levels. The pathological motion in L1 was indicated in red. * The asterisks indicate the motions in the supradjacent levels.

The global flexion, extension, lateral bending, and axial rotation ROMs of the intact and surgical models were shown in Figure 4A. The failure of the L1 vertebra resulted in 7.4, 10.1, 18.0, and 11.5% increases in motion under flexion, extension, lateral flexion, and axial rotation, respectively. All four surgical constructs reduced the global ROM in all directions. The LSPI (U2L2) had the most significant reductions in global ROM in the flexion, extension, lateral flexion, and axial rotation of 88.6, 70.7, 81.1, and 40.7%, respectively. The comparison among U1L1+IS, U1L1+PMMA, and U1L1+Cage showed that TCSR with a titanium cage (U1L1+Cage) results in a slightly larger reduction in ROM than the other two structures (U1L1+IS and U1L1+PMMA), but the differences between each other were all less than 0.5 degrees in all motions.

### 3.3. Motion in the Fractured L1 Vertebral Body and Motion Distributions

Our simulations showed that failure of the anterior and middle spinal column resulted in increased motion in the affected L1 vertebra. The pathological intravertebral motion within the failed L1 was shown in Figure 4B. The largest motion was observed in lateral bending with 0.77 degrees, followed by 0.61, 0.38, and 0.34 degrees in flexion, axial rotation, and extension, respectively. Comparisons between the constructs revealed that TCSR constructs (U1L1+PMMA and U1L1+Cage) had a greater percentage of motion reduction than PI alone. In flexion and lateral bending, U1L1+Cage had the most ROM reduction by 98.6 and 98.1%, respectively. In extension and axial rotation, U1L1+PMMA had the best ROM reduction by 94,9 and 79.1%, respectively.

The ROM distribution was shown in Figure 4C. The pathological motion in L1 was indicated in red and the motions in the supradjacent levels were indicated by the asterisks. In flexion, extension, and lateral bending, all constructs reduced the percentage of motion in L1. Comparisons of the surgical models showed U2L2 had increased ROM distributed in the supradjacent level. In axial rotation, U2L2 had an increased percentage of motion in L1 while other constructs had decreased percentages of motion in L1. The difference in the percentage of the supradjacent ROM in the axial rotation was not significant.

### 3.4. The Effect of PI and TCSR on the Proximal Junctional Level 

**Figure 5:**
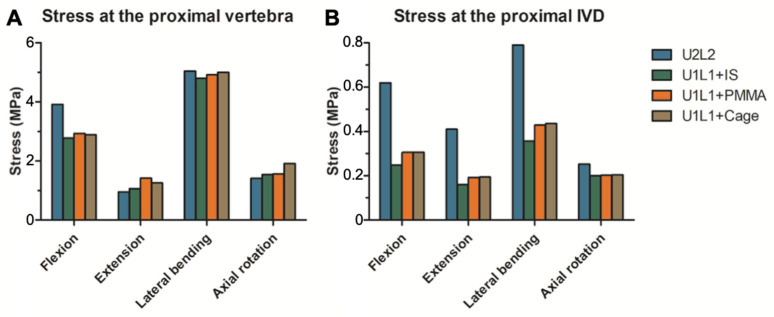
The maximum von Mises stress in the proximal junctional level. The maximum von Mises stress in the proximal vertebral body (**A**) and the proximal junctional IVD (**B**) in flexion, extension, lateral bending, and axial rotation.

The maximum von Mises stresses exerted on the vertebral body immediately proximal to the constructs (T10 vertebra in U2L2; T11 vertebra in U1L1+IS, U1L1+PMMA, and U1L1+Cage) were shown in Figure 5A. For all constructs, the maximum stress at the proximal junctional vertebra ranged from 0.95 to 5.04 MPa. The highest stress occurred in lateral bending (4.80–5.04 Mpa) in all constructs, followed by flexion (2.77–3.81 Mpa). The greatest differences in stress at the proximal vertebra between the constructs occurred in flexion, in which U2L2 resulted in larger stresses by 1.04, 0.92, and 0.88 Mpa than U1L1+IS, U1L1+Cage, and U1L1+PMMA, respectively. The differences in stress at the proximal vertebra in extension, lateral bending, and axial rotation were all less than 0.5 MPa. 

The maximum von Mises stresses exerted on the IVD immediately proximal to the constructs (T10/11 disc in U2L2; T11/12 disc in U1L1+IS, U1L1+PMMA, and U1L1+Cage) were shown in Figure 5B. Comparison between the constructs showed a similar trend in all motions, with U2L2 having the largest stress at the proximal IVD and U1L1+IS having the smallest stress at the proximal IVD. The differences in the proximal IVD stresses between U1L1+PMMA and U1L1+Cage were all within 0.01 MPa. 

### 3.5. The Effect of PI and TCSR on the Proximal Articular Facets 

**Figure 6:**
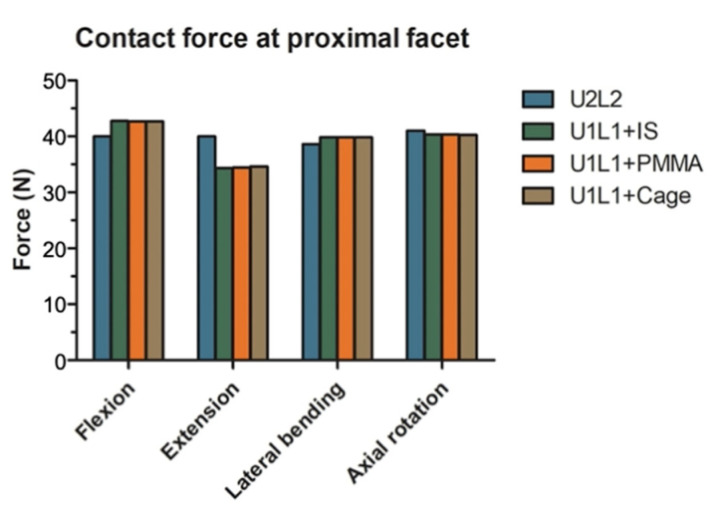
The maximum contact force at the proximal articular facets. The maximum facet contact force in the proximal level in flexion, extension, lateral bending, and axial rotation.

The maximum contact force exerted on articular facets immediately proximal to the constructs (T10/11 facets in U2L2; T11/12 facets in U1L1+IS, U1L1+PMMA, and U1L1+Cage) were shown in Figure 6. In flexion, U2L2 had 2.8, 2.7, and 2.7 N less contact forces on the proximal facet joints compared to U1L1+IS, U1L1+PMMA, and U1L1+Cage, respectively. In extension, U2L2 had 5.6, 5.5, and 5.4 N more contact forces on the proximal facet joints compared to U1L1+IS, U1L1+PMMA, and U1L1+Cage, respectively. The differences in the proximal facet contact forces in lateral bending and axial rotation were all less than 1.2 N. 

### 3.6. Von Mises Stress and Strain Energy Density on the Screw and Rod Construct

**Figure 7:**
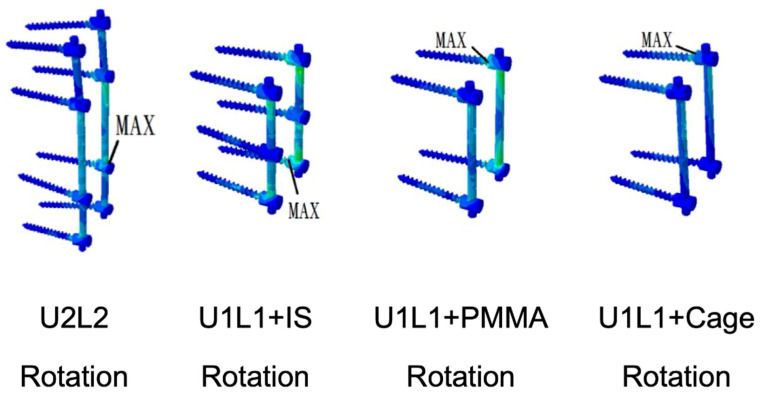
The stress distributions of the pedicle screw and rod constructs in the motions of maximum von Mises stress detected.

The maximum von Mises stress and strain energy density of the pedicle screws in each construct were presented in Table 3. The maximum stress of the pedicle screws occurred in axial rotation in all constructs. U2L2 had the highest pedicle screw stress of 27.98 MPa, followed by 27.31, 24.01, and 16.78 MPa in U1L1+PMMA, U1L1+IS, and U1L1+Cage, respectively. The maximum stress was observed at L2 in constructs involving PI alone (U2L2 and U1L1+IS) but was observed at T12 in TCSR constructs (U1L1+PMMA and U1L1+Cage). The stress distributions were shown in Figure 7. The maximum strain energy density of the pedicle screws occurred in axial rotation in U2L2, U1L1+IS, and U1L1+PMMA, while U1L1+Cage had the highest strain energy density in flexion. U2L2 had the highest strain energy density of 12.41 mJ/mm^3^, followed by 8.05, 5.72, and 4.55 mJ/mm^3^ in U1L1+IS, U1L1+PMMA, and U1L1+Cage, respectively. 

## 4. Discussion

In the present study, we systemically evaluate the biomechanical performance of different TL reconstruction constructs using FE analysis. Our results showed that TCSR constructs provided better stabilization in the fracture of L1 compared to PI alone. Further, there were decreased intradiscal and intravertebral pressures in the proximal level in U1L1+IS, U1L1+PMMA, and U1L1+Cage compared to U2L2. The stress and strain energy of the pedicle screws were lower in TCSR constructs than in PI alone. We showed that TCSR with anterior reconstruction and SSPI provided sufficient immobilization while offering additional advantages in the preservation of physiological motion, a decreased burden on the proximal junctional level, and lower mechanical stress and strain in the implants.

TCSR with PI and anterior vertebral augmentation or intermediate screw fixation has been shown to provide the immediate stabilization and restoration of spinal integrity [2]. Although previous studies have reported the advantages of TCSR in terms of better neurological improvement, stability, restoration of sagittal balance, and fewer implant failures [2,3,4], the rigid constructs in the TL region pose a significant risk for PJF [5], and the ideal strategy for TL reconstruction remains controversial. This study evaluated and compared the biomechanics of different reconstruction strategies using FE analysis.

The T10-L3 FE model in this study was validated against previously published in vitro measurements of the ROM and intradiscal pressure. The majority of our simulation results remained compatible and within one SD compared to the literature [31,32,33,34,36]. Some differences were noted in the intradiscal pressure between our results and previous experiments by Brinckmann et al. (1.24 SD) [35]. Factors such as the anatomical variation between the present model and the cadavers in the literature could result in the differences. Moreover, the location where the intradiscal pressure was measured in the cadaveric experiments could also contribute to the difference since the pressure measured at the periphery of a degenerated disc tends to be greater than the pressure in the center [37,38]. Further, the assumption of isotropic material properties in the present FE model and the difference in the loading application technique might also contribute to the differences since the mechanical responses of the spine to moments in different planes may not be the same. Despite these variations, the difference between our results and that of Brinckmann et al. remained small and within 1.24 SD [35].

To achieve adequate immobilization at the failure level and prevent PJF, the present analysis was aimed to optimize TL reconstruction constructs to minimize motion in the failed L1 level as well as lessen the impact or burden of the constructs on the proximal junctional level. The relation between excessive motion and pseudarthrosis has been established, especially in the TL area, where T12–L2 is susceptible to premature micromotion due to its transitional biomechanics [39,40]. Our current analysis showed that although all constructs successfully reduced the pathological motion at L1, TCSR constructs were shown to provide better ROM reduction compared to PI alone. This is consistent with the clinical results showing better clinical satisfaction, improved fusion rates, and reduced segmental kyphosis in patients receiving TCSR [41]. In addition, the construct of TCSR with SSPI can also provide sufficient stability to the fractured vertebral body, thereby reducing the number of fixed vertebral segments compared to conventional LSPI. As demonstrated in our study and in the literature, this configuration provides the additional advantage of preserving more vertebral motion segments with better physiologic motion and less overall ROM reduction [24].

The present study highlighted the effect of TL constructs on the proximal junctional level by investigating the intravertebral pressure, intradiscal pressure, and facet contact force of the proximal level adjacent to the fixation. PJF remained a significant complication after TL fusion, with associated neurological injury reported in 11-19% of patients [5,42,43]. A major risk factor for PJF was an excessively long fixed spinal motion segment, which is consistent with our results that U2L2 had a higher risk than (U1L1+IS, U1L1+PMMA, and U1L1+cage) [6,7]. We found a reduced intradiscal pressure at the supradjacent disc in all motions and a decreased intravertebral pressure in the supradjacent vertebra in flexion in the constructs with SSPI (U1L1+IS, U1L1+PMMA, and U1L1+Cage) compared to U2L2. It is important to note that although the stimulation of the highest intravertebral pressure occurs in lateral bending, the orientation of the thoracic facet joints and the presence of the ribs and thoracic cage limit the lateral motion of the thoracic segment. Therefore, intravertebral pressure exerted during flexion may be more clinically relevant than lateral bending, so most PJFs are associated with compression and kyphosis in the sagittal plane [5,6].

In addition to investigating the disc and facet joint pressures at the proximal junction near the spinal fixation device for TL burst fractures, this study also investigated the maximum von Mises stress and strain energy of pedicle screws since one of the main problems of SSPI is increased pedicle screw stress, which may contribute to the risk of early implant failure [44]. Since material failure occurs when the von Mises stress surpasses the tensile yield [45], the maximum von Mises stress in the pedicle screws is associated with the risk of acute screw breakage. However, since the tensile yield stress of titanium is approximately 880 MPa and the maximum stress in the present analysis was 29.78 MPa in U2L2, acute screw breakage is unlikely unless there is major trauma. On the other hand, cyclic strain energy during repetitive motion is related to material fatigue, so the strain energy density in pedicle screws may be an indicator of the constructs’ susceptibility to implant failure due to long-term wear [46]. Our result showed that the strain energy of U1L1+PMMA and U1L1+Cage is lower than that of U2L2 and U1L1+IS, while U1L1+Cage has the least strain energy of 4.55 mJ/mm^3^. A plausible explanation for this finding is the effect of stress shielding [47], where part of the axial load is transferred to the anteriorly reconstructed constructs of the PMMA or titanium cage. These results suggested that the TCSR constructs might have a lower risk of implant failure than PI alone and that the titanium cage may provide better stress shielding than PMMA. Further, among the PI constructs, our result also showed that the addition of IS to SSPI also lowers the strain energy density in pedicle screws, but the effect was less compared to TCSR constructs. Taken together, our biomechanical assessments demonstrate that TCSR with SSPI provides adequate stability for an A3 burst fracture at L1 with additional advantages in the preservation of more physiologic motion and reducing the burden on the proximal junctional level to the spinal fixation. Anterior reconstruction with PMMA or a titanium cage also provides stress shielding for pedicle screws, which may lower the risk of screw loosening or wear.

There are some limitations in the present study. First, since the transitional anatomy of the thoracolumbar junction between the rigid thoracic spine and mobile lumbar spine featured unique biomechanics, changing the level of the construct was likely to alter the biomechanical response of the TL segments. With this in mind, considering burst fracture was one of the most common indications requiring thoracolumbar reconstruction, we selected the level with the highest incidence of burst fracture, L1, for simulation [48]. A different location of burst fracture would yield different outcomes in our model. Second, the simplification of the material properties including the assumption of linear isotropic materials might not reflect the real-world behavior of the tissues and the surgical constructs. Third, the position and configuration of the implants including the pedicle screws, PMMA cement, and titanium cage are likely to have variations. Changes in the position and orientation of the implants may vary the motion and stress; however, this is very challenging to simulate since multiple real-world factors including anatomical variation, surgical approach, and surgeon’s preference could all influence the positioning of the hardware. In addition, the bone quality of the spine as well as the decision on whether spinal canal decompression would be performed may also be important issues that affect the overall success of internal fixation surgery. The assumption of the thickness of the cortical bone and endplate might also influence the simulation results. Previous studies have shown that aging and degeneration resulted in decreased endplate thickness [20], and their effect that spinal biomechanics requires future studies to investigate. It should be noted that in the present FE model, convergence tests were performed separately on the spinal model and implant models, and the instrumented model was built based on modifications of the intact model after the convergence tests were performed and the mesh size was reduced. This approach of performing convergence tests prior to the addition of implants was also utilized in previous FE publications [49,50,51,52] and had an advantage in the consistency among the FE models since only part of the model was modified in each surgical construct and the other parts remained unaltered. Finally, perfect contact with tie constraints was achieved between implants and bone. However, the main conclusions of this study were based on comparisons among the surgical construct models. The above-mentioned model simplifications were equally applied to all models, yet their impacts may artificially influence the comparative analyses.

## 5. Conclusions

In this study, we utilized a validated FE model to investigate the biomechanics of different thoracolumbar reconstruction strategies for TL burst fracture and compared their effect on the proximal junctional level. Our results showed that TCSR constructs provided better stabilization in the fracture L1. Further, there were decreased intradiscal and intravertebral pressures in the proximal level in U1L1+IS, U1L1+PMMA, and U1L1+Cage compared to U2L2. The stress and strain energy of the pedicle screws were lower in TCSR constructs than in PI alone. We showed that TCSR with anterior reconstruction and SSPI provided sufficient immobilization while offering additional advantages in the preservation of physiological motion, the decreased burden on the proximal junctional level, and lower mechanical stress and strain in the implants. The knowledge gained from this study can provide help spine surgeons select an optimal TL reconstruction construct to minimize proximal junctional complications.

## Figures and Tables

**Table 1:** Material properties and mesh types of the FE model.

Component	Young’s Modulus (MPa)	Poisson’s Ratio	Element Type
Annulus Fibers			Shell (STRI3)
Inner Laminate: Inner Layer	360	0.30	
Inner Laminate: Middle Layer	385	0.30	
Inner Laminate: Outer Layer	420	0.30	
Outer Laminate: Inner Layer	440	0.30	
Outer Laminate: Middle Layer	495	0.30	
Outer Laminate: Outer Layer	550	0.30	
Annulus Ground Substance	4.2	0.30	Tetrahedron (C3D4)
Cancellous Bone	100	0.20	Tetrahedron (C3D4)
Cancellous Bone (L1 failure)	10		
Cortical Bone	12,000	0.30	Shell (S3R)
Cortical Bone (L1 failure)	1200		
Posterior Bony Elements	3500	0.25	Tetrahedron (C3D4)
Endplate	12,000	0.30	Shell (S3R)
Nucleus Pulposus	1	0.49	Tetrahedron (C3D4)
ALL/PLL/LF/ISL/SSL	20/20/20/10/15	0.25	Truss (T3D2)
Titanium screw/rod/cage	110,000	0.30	Tetrahedron (C3D4)
PMMA	2900	0.30	Tetrahedron (C3D4)

**Table 2:** Element count and mesh type of the present intact model.

Component	Element Type	No. of Elements					
		T10	T11	T12	L1	L2	L3
Cortical bone	S3R	2581	2401	2511	2789	2892	3098
Cancellous bone	C3D4	15,144	17,500	18,509	21,312	23,452	19,079
Endplate	S3R	1905	1780	1796	2145	2010	2268
Posterior elements	C3D4	17,472	16,613	16,820	19,951	20,628	21,503
		**T10/11**	**T11/12**	**T12/L1**	**L1/L2**	**L2/L3**	
Nucleus pulposus	C3D4	4513	3840	3076	5206	4565	
Annulus fiber	STRI3	1025	812	732	1336	1436	
Annulus ground substance	C3D4	5374	4937	3929	6479	5703	
**Ligaments**		**ALL**	**PLL**	**LF**	**ISL**	**SSL**	
**No. of elements**	T3D2	25	25	20	15	10	

**Table 3:** Maximum von Mises stress and strain energy in the pedicle screws.

**Maximum Stress in the Pedicle Screws**				
construct	U2L2	U1L1+IS	U1L1+PMMA	U1L1+Cage
Stress (MPa)	27.98	24.01	27.31	16.78
level	L2	L2	T12	T12
motion	rotation	rotation	rotation	rotation
**Maximum Strain Energy Density in the Pedicle Screws**				
construct	U2L2	U1L1+IS	U1L1+PMMA	U1L1+Cage
Energy (mJ/mm^3^)	12.41	8.05	5.72	4.55
Motion	rotation	rotation	rotation	flexion

## Data Availability

The datasets generated during and/or analyzed during the current study are available from the corresponding author upon reasonable request.
